# Developing an effective care model to empower caregivers to use a humanoid companion robot: an exploratory qualitative study

**DOI:** 10.3389/fpubh.2025.1658136

**Published:** 2026-01-06

**Authors:** Keren Mazuz, Ryuji Yamazaki

**Affiliations:** 1 School of Management, Hadassah Academic College, Jerusalem, Israel; 2 Education Center for Regional Revitalization/Faculty of Glocal Policy Management and Communication, Yamanashi Prefectural University, Kofu, Japan; 3 Symbiotic Intelligent Systems Research Center, Institute for Open and Transdisciplinary Research Initiatives, University of Osaka, Suita, Osaka, Japan

**Keywords:** care model, exploratory qualitative study, humanoid companion robot, human-robot interaction, mild cognitive impairment, reminiscence therapy, robot-assisted therapy, robotic literacy

## Abstract

**Introduction:**

Social robots are increasingly explored as non-pharmacological support for older adults with Mild Cognitive Impairment (MCI) and dementia, yet their day-to-day integration remains limited. This study centers on designing an affective care model that empowers caregivers to deploy a commercial humanoid robot.

**Methods:**

An exploratory qualitative study comprised four observation sessions and ten in-depth, open-ended interviews. A two-phase observational protocol was conducted in a day-care center: Phase 1 mapped everyday activities and baseline interaction patterns; Phase 2 documented two robot-assisted sessions. Detailed descriptions underwent iterative thematic analysis to extract themes and construct a care model grounded in caregivers' practices.

**Results:**

Introducing the robot added care practices that reshaped group dynamics. Two overarching themes emerged: (1) stimulation, engagement, and reciprocity and (2) shared experience and resonance. Together they embody a person-centered, affect-oriented approach that guides caregivers in meeting residents' emotional needs. These themes were synthesized into a structured three-step model, triggering, imagining, responding, that shows how interaction unfolds and how learning is refined through continuous feedback.

**Discussion:**

The proposed care model sequence offers an actionable framework enabling caregivers to transform robotic interaction into a practical, intuitive tool. It provides a new method for training staff to integrate and adapt off-the-shelf robots within routine care, enhancing human-to-human engagement by capturing attention and stimulating memories. Given the global shortage of caregivers, empowering them to run structured robot-assisted sessions offers a scalable, cost-effective solution for health and care organizations working with people living with MCI and dementia.

## 1 Introduction

In recent years, research on social robots for older adults care has expanded rapidly, particularly in supporting daily routines, therapeutic engagement, and the growing needs of people with Dementia and Mild Cognitive Impairment (MCI) (1, 2). Social robots, often known as humanoids, are capable of verbal and non-verbal interaction through autonomous or semi-autonomous sensor-based systems. Studies demonstrated positive effects of social robots on emotional wellbeing, behavioral symptoms, and the quality of life of cognitively impaired older adults (3–8). Yet despite this growing evidence base, a significant gap persists comparatively little is known about how caregivers themselves interpret, adapt, and integrate social robots, especially off-the-shelf robots, into everyday care. This is critical because formal and informal caregivers serve as the primary mediators of interaction and are essential to any successful implementation. Informal caregivers, who comprise much of the hands-on workforce, are particularly overlooked despite their deep experiential knowledge (9).

Although studies show that caregivers acknowledge the potential benefits of robotic systems, they remain hesitant to adopt them due to limited time, technical knowledge, and minimal involvement in customizing or integrating the technology (10). These challenges are intensified by the variability and complexity of care environments, where routines differ widely and commercially available robots often fail to adapt to nuanced, context-specific needs.

One promising direction in addressing these barriers is End-User Development (EUD), which enables care workers to tailor robot functionality based on the practical insights they develop through daily interactions with residents (10). While EUD can enhance contextual relevance, it is insufficient on its own; even user-friendly systems fail when not anchored in existing care models and practices. Caregivers need more than technological tools, they need structured, evidence-based guidance on when and how to use robots meaningfully. Successful implementation must align with what caregivers already do and value, so as not to disrupt care routines that are critical for stability and continuity. Addressing this multifaceted gap, the present exploratory study examines how caregivers interpret robot-assisted therapy sessions and introduces a caregiver-led implementation care model grounded in their routine practices and relational expertise, offering a framework for meaningful, context-sensitive integration of social robots into everyday care.

### 1.1 Affective technology: humanoid robots as non-pharmacological interventions

The rise in dementia and cognitive impairment is strongly linked to population aging, and MCI now represents a critical intermediate stage between normal age-related cognitive change and dementia (11). Each year, approximately 12%−15% of older adults with MCI progress to dementia (12), and many experience Behavioral and Psychological Symptoms of Dementia (BPSD), such as agitation, anxiety, and depression that significantly hinder communication and intensify caregiving challenges. These neuropsychiatric symptoms (NPS) increase caregiver stress (13) and may serve as early indicators of preclinical dementia, including Alzheimer's disease (14).

Against this backdrop, research on social robots for older adults care has demonstrated that continuous interactions with companion robots can enhance mental stability and activity (15, 16), with some benefits persisting even after the robot is removed (17). Humanoid platforms, in particular, are well suited to robot-assisted therapy because their affective design, lifelike gestures, gaze, and expressive voice support the sensory, relational, and emotionally responsive engagement central to non-pharmacological interventions (18). A key mechanism underlying these therapeutic effects is the anthropomorphic design of social robots. Anthropomorphism can generate familiarity, trust, and acceptance (17), and embodied robots elicit higher credibility, empathy, attention, and conversational richness than virtual agents or telepresence systems (4). As Jones (19) argues through the concept of “projective anthropomorphism,” robots function not only as machines but also as semiotic objects onto which individuals project internal states and emotional meanings. These projections can facilitate symbolic dialogue, making humanoid robots particularly effective in emotionally driven care interactions.

Drawing on an exploratory qualitative study conducted in a day care center in Israel, we examine interactions among caregivers, older adults with MCI, and the humanoid robot RoBoHoN. The study asks: What care model can support and empower caregivers in using social robots? Within human-robot interactions (HRI) research, qualitative approaches are essential for understanding emotional engagement, emergent group dynamics, and the projection of human-like qualities onto robots. As Søraa et al. (20) emphasize, more qualitative research is needed to illuminate the social and relational aspects of HRI in care environments. This article proposes a shift from robot-centered to caregiver-centered implementation. Rather than creating new robotic systems, we explore how caregivers can meaningfully use an existing commercial robot as part of their care routines.

## 2 Materials and methods

This exploratory study employed qualitative methods, including observations and in-depth interviews, to understand how caregivers interpret and evaluate group interactions during robot-assisted sessions. The study comprised two phases (as seen in Table 1): Phase 1: observing everyday group activities, how the day care center functions, what activities are available for participants, how the older adults interact with each other and with the caregiver and which care practices are being employed? and Phase 2: introducing a humanoid robot and observing the interactions of the caregiver with the robot and with the older adults.

**Table 1:** Summary of research phases and methods.

**Phase**	**Aim**	**Methods**	***N* (participants)**
Phase 1	Visit 1: Routine activity in the day center	1 Observation (Total observation time: 3 h)	*N* = 40 (34 older adult participants and 6 staff members)
		4 Open-ended interview with the manager and 3 caregivers	*N* = 4
Phase 1	Visit 2: Routine activity in the day center 10 days later	1 Observation (Total observation time: 3 h)	*N* = 40 (34 older adult participants and 6 staff members)
		4 Open-ended interview with the manager, the social worker and 2 caregiver	*N* = 4
Phase 2	Robot-assisted interaction Session 1 (Total session time: 25 min)	1 observation (Total observation time: 3 h)	*N* = 7 older adult participants
		1 Open-ended interview with the manager	*N* = 1
Phase 2	Robot-assisted interaction Session 2 (Total session time: 35 min) 10 days later	1 observation (Total observation time: 3 h)	*N* = 12 older adult participants
		1 Open-ended interview with the social worker	*N* = 1

The purpose of the two-phase structure was to first familiarize the researcher with the center's care routines so that the robot's introduction could be interpreted in direct relation to caregivers' everyday practices. This design allowed caregivers to compare and assess perceived changes in group engagement, emotional expression, and communication. Because the primary focus of the study was on caregivers' interpretations, practices, and meaning-making, the qualitative methods in both phases emphasized in-depth staff interviews supported by observational data. A qualitative, observation-based methodology is well suited for capturing the nuanced changes in group dynamics that occur when a robot is introduced into established care routines. This approach is particularly appropriate for cognitively impaired older adults, who often face difficulties participating in structured interviews or quantitative assessments.

This study operates under the assumption that the presence of the robot facilitates a change in the dynamics of interactions between all participants during the sessions. This change is assessed in comparison to the group interactions and daily activities observed in Phase 1, with the expectation that the robot's introduction in Phase 2 enrich the care practices with new patterns of communication, engagement, and shared experiences. Based on these findings, a care model will be developed. A care model is a structured, theoretical framework that guides how care and therapy are delivered. It draws on findings that identify the core elements of care practices and the caregivers' goals and beliefs about the value of care outcomes.

### 2.1 The study setting

The study was conducted in Israel, in one publicly day care center for older adults with cognitive decline. The day care center serves older adults with functional or cognitive impairments who live at home, promoting independent living for them and their families. This center operates 5–6 days a week, 6–7 h daily (7 a.m.−2:30 p.m.). Activities include morning greetings, breakfast, sports, gardening, music, games, and reminiscence therapy. The center features a main hall, smaller group rooms, and a garden, with secured exits to ensure safety. In the main hall, most of the activities take place using two types of group formation: the first, where participants sit in a circle; and the second, where the participants sit in rows. Eating occurs in small table groups.

The cultural context is central to understanding the center's care practices. This center functions as a community-based facility that provides structured daytime programming aimed at reducing isolation, fostering social engagement, and supporting independent living (21, 22). The center operates within a social-care model that emphasizes autonomy, dignity, and community integration for older adults with dementia and MCI. This cultural context shapes caregiving practices, group routines, and expectations for social engagement, and therefore informs how caregivers will interpret the robot-assisted interactions.

#### 2.1.1 Participants in day care center activities: older adults

All day center's attendants are older adults (age range 65–92 years) diagnosed with mild to severe cognitive impairment or dementia, a key criterion for membership at the center. Eligibility for day-center services is determined through the Long-Term Care (LTC) insurance program administered by the National Insurance Institute^1^. Usually, a functional and cognitive assessment is conducted to determine the level of dependence and their need for ongoing supervision or help with daily tasks due to physical or mental impairment. Those who qualify may attend adult day care centers that offer transport, meals, personal care, therapeutic and social activities, and are overseen by multidisciplinary teams. Thus, participation in a day center indicates that they have already met the LTC eligibility criteria and have been diagnosed with mild to severe cognitive impairment or dementia (No additional screening tools for MCI were administered in this study). The center hosts 30–50 attendees each day, and participation is not mandatory. As a result, different individuals may attend on different days, and group activities naturally include participants with varying cognitive and functional abilities. Consequently, the composition of groups varied across observational sessions. However, all individuals who regularly participate in the center's daily activities and care routines were invited to take part in the robot-assisted sessions.

The robot-assisted sessions took place in the main hall, led by the same familiar caregivers who typically facilitate group activities. Participants were seated in the usual small-group arrangements used during routine sessions, ensuring continuity and comfort. In accordance with ethical approval and to ensure that participants did not feel obligated to join the research sessions, invitations were delivered verbally by the center manager and the social worker both at the beginning of the day and again before each session. They explained the purpose of the study, the voluntary nature of participation, the right to withdraw at any time, and obtained informed consent. Participants were assured that choosing whether or not to participate would have no impact on their access to any services.

#### 2.1.2 Participants in day care center activities: day care center staff

The day care staff include a manager (a woman in her 60s with over 20 years of experience in older adults care and qualifications in education and gerontology), a social worker (a woman in her 50s with over 15 years of experience in older adults care), and six caregivers. A secretary and a maintenance worker are also part of the staff. The caregivers, most of whom are women, provide companionship as well as personal and social care, including assisting with daily activities and helping to organize group sessions. All staff members are between 50 and 68 years old and are trained in the care of people with MCI and dementia. Although staff members use computers and applications in their administrative work, they have no prior experience interacting with social robots, and the center does not incorporate technological or digital tools into its daily activities.

#### 2.1.3 The humanoid companion robot

This study utilized Sharp Co.'s RoBoHoN SR-05M-Y in its off-the-shelf, commercially available version, without any technological modifications^2^ This robot is designed to sit and move its arms and head autonomously using servo motors. It supports speech interactions in English and Japanese via speech-to-text (STT) and text-to-speech (TTS) servers. The robot's expressive behavior was conveyed through its voice-recognition system, speaker, LED lights in the eyes and mouth, and coordinated arm and head movements^3^.

For this study, RoBoHoN operates as a rule-based AI system that follows predefined commands only in English, such as “dance,” or “sing.” Caregivers communicated with the robot exclusively in English and were instructed to use only these available voice commands. The robot responded verbally only in English with short phrases such as “Dancing is so much fun” after completing a dance or when introducing a song before beginning to sing. Some of its songs are preprogrammed in Japanese and are selected autonomously, so it was not possible to predict in advance which language would be activated. Some of the older adult participants were able to understand and imitate the caregivers' instructions and independently issued the English commands “dance” and “sing.” These were the only English words they used, and they were simple, intuitive, and easy to follow. All interaction between the caregivers and the older adults took place entirely in Hebrew.

### 2.2 Observational study methods

This exploratory qualitative research comprised of 4 observation sessions and 10 in-depth open-ended interviews. After every observation, interviews were held so caregivers could reflect on their subjective experience of the interaction and on what had been observed (interviews were conducted with the manager, the social worker and caregivers). As seen in Table 2, the observations were structured to explore three aspects of the physical, visual, verbal, and non-verbal interactions of the participants toward the robot, the caregiver and between the participants.

**Table 2:** A summary of the observed aspects.

**Observed aspect**	**Guiding questions**
Communication message	What was the content of the conveyed messages?
Shared experience	What was the shared content between all participants in the “here and now” of the interactions?
Communication methods	How were the messages conveyed?

#### 2.2.1 Observation procedures

In phase 1, two observations were conducted on two different days (10 days apart) along the day (between 9 a.m.−14 p.m.). Group activities were observed to see how the older adults interact with each other and with the caregivers and staff members. The observer sat in the main hall where the activities took place but at some distance from the group and did not participate in the activities.

In phase 2, two robot-assisted interaction sessions scheduled at midday (between 12 p.m.−14 p.m., after meals) were observed, noting the interactions of the older adults with the robot, with each other and with the caregiver. Taking into consideration the communication profiles of people with MCI admitted to this center, these sessions were based on a bottom-up communication format that resembles the “free association” method of therapy. In this format, participants (older adults and staff members) may spontaneously say and communicate anything that comes to mind when engaged with the robot. This enables them to communicate words, thoughts and emotions through a more “freely” associative flux in intersubjective shared interactions (23).

In the first session, the day-care manager joined the group, while in the second session the social worker participated. Both were asked to engage naturally and to “go with the flow” of the interaction, following the free-association approach in which older adults could speak spontaneously and move between topics without predetermined structure. The sessions were conducted in Hebrew and each lasted 20–35 min. Different participants attended each session.

In both sessions, the robot was placed at the center of a table in the main hall, with chairs arranged around it. It was connected to a power outlet and wireless Internet. At the start of each session, the manager introduced the research and the robot to the group. In the first session, the manager sat behind the robot alongside the researcher, who operated the robot in English, while participants both invited and spontaneous gathered around the table. In the second session, the introduction was followed by the participation of the social worker and a caregiver, who joined the group interaction around the robot.

The interviews were conducted after every observation with the center's staff to explore their interpretations and perspectives by asking: What do you value most in the care you provide? What does “care” mean within the context of your daily work at the center? In what situations would you consider using a robot? How did the robot-assisted sessions compare with your regular daily sessions in terms of group engagement, emotional response, and communication? Would you feel comfortable leading or facilitating a robot-assisted session? Why or why not? Through these questions, we aimed to better understand how caregivers relate to the care practices, values and experiences.

During the sessions, the researcher documented all verbal and non-verbal interactions of the participants, categorizing them into three aspects described in Table 2: communication messages, shared experiences, and communication methods. No audiovisual recordings were made. Instead, detailed reflexive field notes were documented immediately after each observation and interview, then transcribed and translated into English.

### 2.3 Data analysis

Findings are presented through a bottom-up thick description of the observations. A thick description is an ethnographic rigor strategy that enhances the transferability of qualitative findings by situating behaviors within their social and contextual meaning (24). In this study, thick description served to integrate interviews, observations, and field notes into a unified interpretive account, capturing the phenomenological “here and now” of interaction and enabling an in-depth understanding of subtle behaviors, emotional responses, and interpersonal dynamics. This triangulation was particularly important because participants with cognitive impairment do not always verbalize their experiences; field notes therefore documented meaningful non-verbal behaviors such as stroking the robot, smiling at it, or softly speaking to it, allowing these responses to be contextualized alongside verbal accounts. The researcher conducted open coding to identify descriptive units related to interactional behaviors, emotional expressions, and caregivers' interpretations. Codes from routine sessions (Phase 1) and robot-assisted sessions (Phase 2) were systematically compared to identify contrasts and shifts in interactional patterns. Through iterative thematic analysis and comparison, codes were clustered into subthemes that reflected recurring caregiving strategies and interactional dynamics^4^. These subthemes were then integrated into two overarching themes: Stimulation, Engagement, Reciprocity and Shared Experience and Resonance (as seen in Table 3).

**Table 3:** . Summary of the themes, subthemes, and comparisons between routine (Phase 1) and robot-assisted (Phase 2) sessions.

**Themes**	**Subthemes**	**Codes from Phase 1**	**Codes from Phase 2**
**Stimulation, Engagement and Reciprocity**	• Individualized interaction strategies • Emotional sensitivity and presence	• Personalized approach (i.e., calling participants by name, keep eye contact) • Music as memory stimulus *(quote:* “Music to evoke reactions and memories”).	• Relational naming and anthropomorphizing *(quote: “*the robot looks like my grandson… we'll call him *Moshe*”). • Projecting care onto robot *(quote:* “Is he alone? Where are his parents?”).
**Shared experience and Resonance**	• Mutual focus • Physical and emotional mirroring • Eliciting memories and sharing personal and family narratives	• Fragmented attention *(quote:* “Participants respond freely, sometimes speaking over one another…”). • Lack of group cohesion *(i.e.*, there are no shared goals or outcomes).	• Joint physical movement *(quote:* “They observed each other's movements… all followed the robot's exercise.”). • Playful mimicry *(quote:* “She stuck out her tongue… immediately the others did the same”). • Spontaneous storytelling *(quote:* “She spoke about her parents, siblings, and children… the group laughed”).

Triangulation across observations, interviews, and reflexive field notes strengthened the analytic process and supported the reliability of the findings. Throughout the analysis, the researcher engaged in reflexive memo writing to examine positionality, assumptions, and potential biases, ensuring transparency in the interpretive process. Themes were developed inductively and refined through close engagement with caregivers, who contributed to interpretation by contextualizing behaviors, clarifying routine practices, and commenting on the emerging structure of themes. Following Mazuz and Yamazaki's (25) analytic procedure, each theme was tracked across sessions to determine whether it was present, absent, or only partially evident. For example, caregivers expressed a desire for a shared group experience, which was largely absent in Phase 1 but emerged spontaneously during robot-assisted interactions, underscoring its significance within affective care. All observations and interviews were conducted by a single researcher who had prior familiarity with the site. This insider position was explicitly acknowledged and addressed through structured reflexive notetaking and attention to potential expectancy effects.

After completing the thematic analysis and constructing the care model, the model was interpreted through psychoanalytic object-relations theory to illuminate its therapeutic potential. Although psychoanalysis has addressed dementia only to a limited degree, object-relations theory offers a valuable framework for understanding emotional dynamics, relational needs, and internal psychological states in individuals with cognitive decline^5^^.^ Evans (26), for example, provides a psychoanalytically informed approach that guides caregivers in recognizing and responding to the emotional suffering experienced by people with dementia, from early MCI to advanced stages.

## 3 Results

The analysis was organized by two clustered themes as seen in Table 3, integrating detailed quotes and comparisons between routine (Phase 1) and robot-assisted (Phase 2) sessions. Each theme represents a core value of care practice that forms an integral part of the person-centered approach to providing affective care.

### 3.1 Theme: stimulation, engagement, and reciprocity

During the morning sessions (8:30–10:00 a.m.), participants sat in a circle with background music while a caregiver engaged with each person individually. These sessions typically began with staff greeting each participant, although participants themselves did not formally greet one another. When the caregivers see older adults losing interest, they work in small groups where they can stay close, maintain eye contact, or give a gentle touch to regain attention. The activities must be easy to change immediately: if exercise or music fails to engage, they simply switch songs or movements.

In other group sessions led by an occupational therapist, the participants sat in two rows, engaged in cognitive and memory exercises. The therapist, standing at the front, moves across the room keeping eye content and draw the attention of the participants by calling their names or loudly vocalized her memory-test questions. Participants respond freely, sometimes speaking over one another, maintaining focus on the therapist. During this task-oriented exercises the therapist avoids forcing a single correct answer or one solution, because participants move at different paces and respond in different ways.

Engaging participants in activities observed in Phase 1 such as games, music, movement, and gardening requires capturing their attention, often through tactile or direct stimuli. The staff use personalized approaches, including face-to-face communication, making eye contact while calling participants by name, using nods and encouraging verbal and non-verbal intonations (i.e., uh-huh, yes, go on..), and using music to evoke reactions.

Even during group activities, they prioritize individual interactions to stimulate responses and maintain engagement. As the center manager explained, the team “tries their best to become emotionally attuned to each participant's needs so that each of them has a good day.” She also noted that, given limited staffing, a participant who becomes agitated or aggressive often requires one-to-one support, which means a caregiver must step away from the group. To keep the session running, all caregivers need to be familiar with the activity plan so they can step in and assist whenever needed. This is one reason why group activities are planned and preferred; they allow flexibility while still enabling individual attention. In their approach, “care” and “entertainment” are not separate categories. Any activity that a participant draws in, whether it is a seated exercise, singing a familiar song, or dancing, becomes therapeutic the moment it triggers engagement, sociality, emotional connection, cognitive stimulation, and physical wellbeing^6^.

The caregivers explained that they assess, and value care outcomes based on a feedback from the older adults and their families, though this feedback is often subtle or indirect. As a result, shared expectations among staff have usually focused on observable indicators: that participants remain calm and not agitated, express their feelings to some degree, attend the center regularly and stay for the full day, avoid crying, maintain personal hygiene, eat well, and take part in activities, even when the outcomes or engagement are not always clearly visible.

However, these efforts to engage the participants and to create reciprocal interactions were challenged by limited capacity of staff and participants' cognitive barriers. In the interviews, staff described difficulty “accessing the inner world” of the participants, highlighting the limitations of verbal or task-focused engagement. Caregivers agreed that reminiscence is a valuable tool to trigger memories and to encourage participants to share information, yet suitable moments for it are not always available.

In Phase 2, the robot introduced a new stimulus that captured the participant's attention and elicited spontaneous group responses and interactive dialogue with the robot and among the participants. When the manager of the day center introduced the robot and asked it to dance and sing, seven older adult participants, some invited and others joined spontaneously, gathered around the robot, focusing their attention on the robot. Some participants became curious and inquired about the robot's name. One participant suggested “*Moshe*,” remarking that the robot resembled their 10-year-old grandchild. The other participants followed him and began referring to the robot as *Moshe* asking with concern “Where are his parents? Is he alone?” Others keep asking, “Where does he live? Can I touch him? How much does it cost? Where is it from? Can it dance again? Can he walk or stand?”

Engaging with the robot included verbal and non-verbal involvement (e.g., singing along, copying the robot's actions, asking questions). The manager was struck that the participants had talked about their grandchild in the group.

The robot then started to perform a seated exercise while singing and moving his arms and head. All the participants including the manager, followed the exercise, humming along to the music and moving their hands and heads. They looked at each other and observed each another's movements. This exercise routine lasted approximately 30 s, during which the robot continued to sing and dance and ended saying “dancing is so much fun!,” the participants applauded the robot for its performance. The robot sessions prompt a noticeable rise in interactions compared to routine sessions, reflecting higher levels of stimulated participation. The caregivers highlighted the value of well-sequenced activities: everyone should know what starts now, how it unfolds, and when it ends. So for example when the robot asks at the end of a dance “dancing is so much fun!,” it is important to completes the activity.

In another session, one of the woman participants after engaging with the robot asked loudly: “Why is he sticking out his tongue?.” She then protruded her tongue to the robot, and immediately, all the other participants who sat next to her did the same, looking at each other until the participant who started this stopped. The manager smiled at the participants, struck by and enjoying these interactions. She responded loudly to all of the participants, partially questioning and partially agreeing “He is sticking his tongue” (referring to the robot as a little boy in Hebrew) and the woman participants said “Yes, he is just a *Yald*” (“just a little boy” in Hebrew). In this interaction, the robot looked human, referring to the LED lights forming the robot's mouth as if it is a tongue. This interaction was unique in its reciprocity that was observed in the turn-taking, and the playful exchanges between the participants.

This session exemplifies reciprocal interaction through a layered, dynamic exchange between the participant, the robot, other group members, and the caregiver. The other participants immediately imitate the woman that protruded her tongue to the robot, indicating peer-to-peer engagement stimulated by the robot's behavior. This forms a loop of response and mimicry among the participants. The participants were interested not just with the robot but also with each other. The manager's verbal acknowledgment integrates her attention into the loop, reinforcing their behavior. Her enjoyment signals that the interaction is both meaningful and endorsed. The participants' interpretation of the robot as a “little boy” adds a relational and emotional frame.

### 3.2 Theme: shared experience and resonance

During the routine sessions observed in Phase 1, the participants sat in a circle or rows, their attention remained focused on staff, not each other. Although participants were seated in a circle, there was minimal bonding or peer dialogue. For example, in the morning sessions, the older adults do not particularly greet or relate to each other or form bonding friendships, or during a memory exercises the focus was on the cognitive game so each participants respond freely, sometimes speaking over one another. While valuable, these interactions were with little peer dialogue, and responses were often fragmented, as described by one of the caregiver, “there are no shared goals or outcomes fostering communication or interaction.” The participants primary interactions were with the staff rather than with one another, this group activities exhibit low levels of entitativity and cohesiveness among participants (27).

As the caregivers described, in shared musical activities the participants sing or move to the rhythm, creating a visible sense of shared engagement. This synchrony is perceived as a form of connection. Similarly, during physical activities or gardening, participants tend to follow instructions, even if each does so at their own pace. In contrast, memory games reveal noticeable gaps between individuals, making it difficult to have a shared experience. When a participant appears disconnected from the group, caregivers actively intervene, gently supporting them in reorienting and rejoining the group environment.

In Phase 2, the robot elicited joint attention and synchronized group behavior. During a seated exercise led by the robot, all participants moved their hands and heads to the robot singing and dancing, watching each other. As exemplified above, when one woman though the robot protruded his tongue, she did the same and others mirrored her, creating an unexpected moment of a shared experience. Participants are not just reacting individually but reflecting and amplifying each other's emotional and behavioral cues through resonance.

In one interaction, while the robot is singing and dancing, a conversations started between an older adult woman and the social worker. The woman said: “I will ask my son to bring me one…from where it is? Can I bring one?” The social worker responded: “It is from Japan. Can you get one?” The woman said repeatedly: “I will ask my son to bring me one. He works all the time, he works abroad.” The social worker asked her “What will you do with it?” The woman replied, looking directly at her, “I will play with it.” She then spoke directly to the robot, asking, “Would you like to come to my home, little boy? You are cute like my son.” While the robot sang, the woman began sharing her story about her family, maintaining eye contact with both the robot and the social worker. She spoke about her parents, siblings, and children, humorously recounting her son's close relationship with his sister, which often made the son's wife, a policewoman, jealous. The group was attentive to her story.

After the robot finished dancing, no one requested that it dance again so the robot turned its head toward the participants' voices, lighting up its eyes in green and white, and adopting a posture of listening to a conversation that it cannot follow (i.e., hunching its shoulders and tilting its head from side to side, as if trying to understand).

In respond to the woman story, the social worker then shared her own story about a stressful encounter that happened to her with a policewoman while waiting for her own daughter. The participants empathized, voicing their support and engaging deeply in the discussion. This moment of shared empathy and connection, initiated by curiosity about the robot, encouraged open conversation about family and motherhood. As the dialogue continued, the woman expressed her love for the day center and its caring environment. She leaned in toward the robot, placing her hand next to the robot, listening intently as it sang. The participant story prompts the social worker to reciprocate with her own narrative. This back-and-forth reminiscence exchange creates a moment of shared resonance.

The social worker later reflected on the session, noting the unexpected depth of reminiscence that emerged, not only from the participants, but also from her own story. She emphasized that she had not intended to lead a reminiscence session, it happened spontaneously. Although she and other staff members sometimes avoid certain topics to prevent emotional distress for participants, this session unfolded differently. Drawing on her previous experience with one-on-one reminiscence, she observed that this group session engaged more participants, especially around shared themes like motherhood and family. She felt that because the robot served as the focal point, it became easier for both the participants and herself to open up. The focus shifted away from them as individuals and toward the robot, creating a more comfortable space for sharing. On reviewing the conversation afterward, she reflected that it is common for participants to respond to memories with a range of emotions, both positive and negative. In this case, despite the personal stress in her story and the woman's sadness about her children living far away, the overall tone was positive. The woman even ended the session with kind words toward the robot and about the center.

The social worker recommended using the robot as a companion in reminiscence therapy, emphasizing that it should be able to recall past sessions, retrieve images and topics based on emotional responses, particularly negative ones, and provide real-time updates to caregivers. This functionality would allow staff to track changes in a participant's emotional state, improve coordination among caregivers, and enhance the overall quality of care.

In other interviews, caregivers expressed that they would feel confident using the robot only if they fully understood its capabilities and had a clear strategy for when and how to use it, including what to expect during sessions. They stressed the importance of receiving immediate verbal and non-verbal feedback from the robot to help them respond in the moment and detect subtle changes in the interactions with the participants.

## 4 Discussion: the care model feedback loop sequence

This study operated under the assumption that the presence of the robot facilitates a change in the dynamics of interactions between all participants. Findings reveal the impact of the robot on the dynamics among the older adult's participants and their interactions with the caregivers. The robot initiated a change, a shift in the group dynamic. It stimulated interaction; woke up the group; evoked a memory. Social robots are known to indirectly influence group behavior, causing a “ripple effect” (28), meaning that robots influence the interactions that people in the group have with other people (i.e., human-to-human interactions).

As a consequence of the presence of the robot, numerous interactions and communications emerged that had not been present before (in relation to Phase 1). Two interconnected effects emerged that highlight the care practices: an increase in amplitude of the interactions (a scale up, more interactions and communications), and a reciprocity among interactions.

From the observer's perspective, the verbal and non-verbal communication after the introduction of the robot appeared coherent. There were no delays in response, no distractions from the main theme; people were engaged. Their attention was focused. Their conversation was pointed. Their thoughts and reasoning seemed entirely coherent in context. A conservational exchange took place, and it was clear that all participants understood each other. These observed communications revealed responsiveness and emphatic resonance among all the participants: they were talking to each other, making eye contact, and conversing deliberately and pointedly with the robot and the caregiver. They stayed in physical proximity, and one woman even came right up to the robot. Their interactions were in sync, resonating in a shared experience. The caregivers set an example for others, encouraging an environment that promoted empathic resonance—an emotional connection and shared experiences among the participants. Empathic resonance involves individuals attuning each other, understanding and relating to each other's emotions, thoughts, creating a sense of connection and shared experience.

The themes described above, such as, stimulation, engagement, reciprocity, shared experience, and resonance, capture the core care practices observed in both routine and robot-assisted sessions. Together, they embody a person-centered, affect-oriented approach that guides caregivers in meeting residents' emotional needs. These practices comprise a structured care model that shows, step-by-step, how interaction unfolds and how learning is refined through continuous feedback.

**Figure 1:**
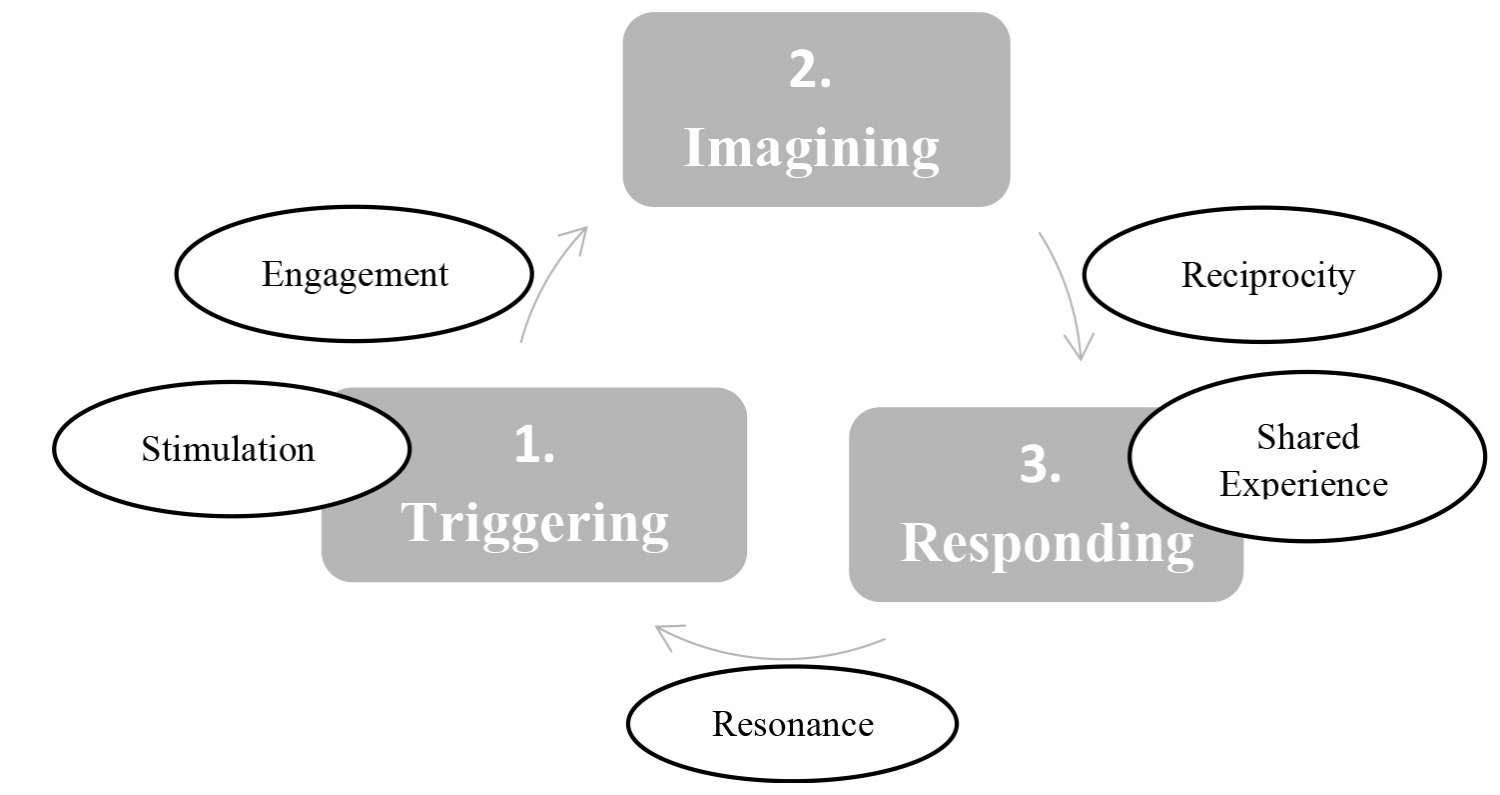
The care model feedback loop sequence.

The care model progresses through three interlocking stages: triggering, imagining, and responding (see Figure 1).

**1 - Triggering:** Each session at the center began by capturing the participants' attention through stimulation (i.e., call each person by name, maintain eye contact, use music). Also the robot captures the participants' attention through sensory stimulation. It has human features: lights as eyes, a voice to talk and sing, it can move its head and arms, and dance. The caregiver and other staff members, in their modeling role, stimulate the participants' curiosity – asking questions, touching the robot, and becoming excited. The robot's singing and dancing repertoire serves as a non-linguistic cue that resonates and further stimulates engagement and collaboration through oscillatory relationships (29). Participants responded to the robot's singing in different languages without needing translation, engaging physically with its rhythm and overall vibe. The robot is designed with humanoid features that stimulate a reaction or evoke a memory. Humanoid development in Japan is focused on stirring human reactions and “sense engineering” (*kansei* or affect engineering) (30).

**2- Imagining:** Once the participants is already engaged, their attention is sustained. Through the robot-assisted session, participants engage in anthropomorphizing and make-believe (i.e., a suspension of reality by attributing human-like intentions, emotions and behaviors to the robot and acting as if the robot is a little boy or participants interpret the red light illuminating its mouth as protrusion of the tongue). In other sessions, the initial trigger to engage results in reciprocity that continues and amplifies into engaging conversation with others.

While we acknowledge that novelty may contribute to the participants' engagement, the unique anthropomorphic effects of the humanoid robot must be considered. Jones (19) explains projective anthropomorphism as an unconscious bias toward anticipating humanlike characteristics in robots, “Robotic creatures become enhanced in their capacities to enact scenarios in which robots are Rorschachs, projective screens for individual concerns” (p. 2066). Thus, anthropomorphic motifs attributed to social robotics “are emergent properties of a tacit dialogue with our “inner” selves, collectively as well as individually” (19, p. 2063). These imaginings and reflections may be consciously or unconsciously felt, echoed, fantasized, and contemplated leading to inter-subjective and shared sense-making through words, gestures, and behaviors. Participants projected human-like characteristics onto the robot (e.g., “Moshe,” “a little boy”) and responded dynamically with specific behaviors (e.g., synchronized actions, shared laughter, and conversation about family). Personal objects, such as family photos, artwork, and religious symbols have been shown to prompt reminiscence effectively (31), as have tailored sensory stimuli, like familiar music, videos, books, and newspapers (9). When the robot is anthropomorphized, it too can act as a personalized cue, serving the same purpose. These interpersonal connections suggest more than a mere novelty response and align with psychoanalytic theory, particularly the projective anthropomorphism.

**3- Responding**: Participants respond to the caregivers' activities as well to the robot's dancing and singing by clapping their hands, dancing, and singing themselves. As opposed to the routine sessions, in the robot-assisted session, they also respond to each other in relation to the robot's activities, paying attention to social norms and cues, such as protruding the tongue. The participants look at each other more often. The conversation between the woman and the social worker triggered by the perception of the robot as a little boy and resonance a conversation about motherhood, family relations, and protection. During the conversation, the woman and the social worker responded intuitively and viscerally to each other, in a shared experience. The robot's change in posture as the conversation continues and its eyes light up and head tilts unable to follow the conversation, triggers further and closer interaction as the woman approaches the robot with concern. As she continues “I love this place” there is a profound insight into her thoughts and feelings about the day center.

### 4.1 Analyzing the therapeutic effects of the care model

From a psychoanalytic object-relations perspective, this care model involves dynamic interplay between intra-subjective (intra-personal) moments where each participant imagines, and inter-subjective (inter-personal) moments where the participants' attention is triggered, and responses are made to each other. While the first and third stages—triggering and responding—are manifest through verbal and non-verbal communication and observable behaviors with others, the second stage—imagining—remains somewhat elusive where unconscious processes occur within the inner world of each participant.

In psychoanalytic terms, the participants' conversation with the social worker reflects a container/contained relationship (32). This undermines the potential for “reverie” (33) as described by Bion (32) “tolerance of the experience of being adrift” (p. 569) or the “dream work” as discussed by Ogden (34). This “reverie” facilitated an environment where the thoughts and feelings derived from the participants' lived experiences were expressed and processed (i.e., anthropomorphizing the robot). For example, the participants' conversation and reactions to each other reveal that they have connected in their perception of the robot as a little boy, as they assume maternal and parental roles in emphatic resonance.

This care model sequence enables “adaptive learning,” eliciting varying reactions from humans who encounter the robot and try to make sense of the experience (35). This model may facilitate a robot-assisted therapy sequences to be used by caregivers. In psychoanalytical therapy terms, the human-robot interactions is an interval that has a transitional quality of a “potential space, the third area of experiencing,” as described by Winnicott (36, p. 1355).

Along the sequence as described in Figure 1, there is a dialectical interplay between reality and fantasy that stimulates imagination, make-believe and play. According to Winnicott (36), playing happens within a potential space and mediates between the real world and the playing self. This potential space is an intermediate area of experiencing that lies between the intra-subjective world (or inner psychic reality) and the intersubjective world (actual or external reality).

This is particularly significant for individuals with MCI, where communication is often implicit and linked to memory challenges. Sobral et al. (37) found that older adults with MCI have reduced abilities to sustain conversations or articulate their emotions and needs, leading caregivers to misinterpret their communication as solely reminiscence or memory recall. Thus, a care model grounded in the caregivers' practices is important. Care staff interviewed in this study positively evaluated interactions between the humanoid robot and participants, highlighting the humanoid robot as an assistive companion because of its benefits and potential.

### 4.2 Practical implications

Table 4 translates the care model into an everyday strategy and roadmap, showing caregivers where familiar routine practices of reminiscence, for example, meet the new possibilities offered by a social robot.

**Table 4:** Integrating the care practices themes (Stimulation, Engagement & Reciprocity, and Shared Experience & Resonance) across the care model sequence (Triggering–Imagining–Responding) with routine vs. Robot–assisted reminiscence session.

**Stage**	**The care practices themes**
**Triggering**	**Stimulation** • Caregiver presents a photo, song, scent, or personal object to evoke memories. • *Robot can greet by name, display the exact photo or play the song on cue, adjusting volume/lighting automatically*. • Open-ended questions invite personal stories; caregiver mirrors facial expressions, validates feelings. *• The robot reacts in non-verbal cues of thinking or plays background sounds to widen the sensory context*.
**Imagining**	**Engagement & Reciprocity** • Caregiver watches eyes, posture and tunes the stimulus if residents seem agitated. *• Robot's facial recognition or voice-stress detection flags emotional responses and alert the caregiver*. • Caregiver listens actively, asks questions and encourages more images to imagen, participants respond, sometimes to each other. *• Robot's dialog manager notices pauses and gently re-engages the residents, freeing the caregiver to note subtle affect shifts*.
**Responding**	**Shared experience & Resonance** • Caregiver summarizes the stories, offers empathic praise, and documents preferences for next time. *• Robot delivers immediate positive feedback (“That memory made all of us smile!”) and logs who engaged, for how long, and with what trigger*. • Caregiver soothes any residual sadness, thanks participants, and checks how they feel. *• Robot detects negative effects and can switch to a calming animation or breathing cue, while caregiver gives tactile reassurance*. • Group members echo each other's memories; caregiver highlights common threads. *• Robot pulls up another resident's related photo in real time, letting stories intertwine and reinforcing group cohesion*. • Caregiver links the stimulus to a group theme. *• Robot projects the image on a shared screen so the whole group circle leans in together*. • Caregiver points out shared laughter or insight, reinforcing group identity (“We all had a good memory today”). *• The robot replays a brief “highlight reel” of session photos or words, giving everyone a collective closure*.

By aligning each stage (i.e., Triggering, Imagining, and Responding) with the continuous goals of stimulation, engagement, and shared resonance, it demonstrates how the robot can join the caregivers' practices. In doing so, the model answers the research question: it guides caregivers to weave robotic functions into tasks they already perform and value, while promoting affective care qualities of attunement, reciprocity, and shared meaning. The result is a practical, step-by-step framework that empowers staff to use social robots with cognitively impaired older adults, ensuring that technology enhances rather than disrupts the daily care.

### 4.3 Research limitations and future directions

The three-step “*Triggering–Imagining–Responding”* heuristic in this article offers staff a practical guide to spotting engagement opportunities and guiding interactions without any custom programming. However, this study was designed as an exploratory observational study, and the care model developed here is in need of further empirical validation. Several potential confounding factors must be taken into consideration in future research.

A primary limitation concerns the potential novelty effect when participants may have shown heightened enthusiasm simply because the robot was new and unfamiliar. Although baseline observations of routine activities were conducted in Phase 1 to contextualize behavioral changes, the short duration of exposure means that some of the observed engagement may still reflect novelty. This applies not only to participants but also to caregivers, whose curiosity and expectations may have affected group responses. However, within the care model developed in this study, novelty is not treated solely as a confound but also as an intentional component of the “Triggering” phase. The first stage of the model sequence relies on capturing attention through stimulation, whether through familiar cues (e.g., calling a person by name, using music) or unfamiliar ones (e.g., the robot's movements, lights, or singing). Novelty thus plays a functional role by helping to initiate engagement and focus at the outset. According to the model, even if the novelty effect diminishes over time, caregivers still need to trigger attention, and this can be achieved through other sensory or interpersonal cues once the robot becomes familiar.

Future research should therefore examine how the care model operates beyond the initial novelty window. Longitudinal studies with repeated robot-assisted sessions could explore whether the “Triggering” phase continues to elicit engagement once the robot is no longer new, and whether the subsequent “Imagining” and “Responding” stages are maintained, deepen, or change in quality over time. Comparing early and later sessions, as well as contrasting the robot with other non-robotic stimulus, would help distinguish between short-term novelty-driven responses and longer-term adaptive learning and therapeutic effects within this care model.

Longer-term observation would also reduce the impact of environmental familiarity and observer effects, as participants become accustomed to both the setting and the researcher's presence. Finally, extended data collection with multiple sessions and blinding procedures would enable more robust reliability checks, strengthening the validity of the care model's outcomes. Future research should incorporate the older adult's participant's interviews to deepen understanding of their perspectives about the care practices.

Moreover, the robot's inability to communicate in Hebrew represents an important technical limitation, as RoBoHoN operated only through English voice commands and autonomously selected songs in English or Japanese. While using off-the-shelf robots is standard practice in early-stage HRI research, the language constraint may have restricted the depth of interaction and should be addressed in future studies through teleoperation or customized AI-conversational modules.

Future studies also could explore the care model feedback loop sequence described here using commercially available off-the-shelf robots. This approach could offer scalable, cost-effective solutions, enabling broader access to robot-assisted care in health and care organization. The robot-assisted interaction sequence may vary depending on context and different robotic platforms. Using a teleoperated robot system (by Wizard-of-Oz setup) or through End-User Development (EUD) application (10) may provide more contextual data on cultural content and methods of communication between caregivers and older adults, enabling engineers to customize conversational scripts.

Another critical consideration is the impact of robot-assisted therapy on caregivers themselves. Perhaps the most useful effect was among the caregivers. Beyond simply facilitating sessions, caregivers played an active role in modeling interaction with robots, encouraging participation, and co-creating shared experiences with the older adults. This highlights the importance of robotic literacy, that is, the caregivers' ability to understand, interact with, and effectively incorporate the robot into their routine practices. To ensure that the three-step “Triggering–Imagining–Responding” heuristic could be meaningfully applied, strategies were used to support and assess caregivers' engagement during sessions, including unscripted interaction formats, familiar staff involvement, and post-session reflections. These approaches revealed how caregivers intuitively aligned with the care model, using the robot not as a replacement for their role, but as an extension of their therapeutic toolkit. Nevertheless, future research should more systematically explore how exposure to robotic platforms affects caregivers' attitudes, communication strategies, and sense of efficacy in supporting individuals with cognitive impairment. Evaluating and enhancing robotic literacy among care staff will be essential to successfully scaling such interventions in diverse settings.

To establish robust clinical outcomes for the care model using a robot-assisted therapy, future research should include quantitative measures of cognitive improvements or retention effects. For instance, short interventions, such as a 5-min RAA-CR session (6), could provide measurable cognitive data. By addressing these directions, future research can refine robot-assisted interventions based on the care model, uncover their full therapeutic potential, and offer scalable, culturally adaptable solutions to enhance the lives of cognitively impaired older adults and their caregivers.

In addressing methodological considerations, it is vital to balance quantitative and qualitative approaches. As noted by Flandorfer (38), qualitative studies are more flexible in uncovering new psychological or social factors, while quantitative methods often rely on predefined scales, which can limit the exploration of novel insights. Employing participatory, user-centered design methods (20) or individualized technology approaches (38) can place greater emphasis on the unique (socio)psychological experiences of users. Combining qualitative and quantitative methods through triangulation would further strengthen the understanding of robot-assisted therapy and its outcomes.

From a psychoanalytic perspective, future research could investigate whether humanoid robots function as “transitional objects” (36), providing emotional support in the absence of caregivers. This concept is particularly relevant for individuals with cognitive impairments or dementia, who often experience feelings of loss, separation, and insecurity (39, 40). Understanding the role of robots as attachment objects may reveal their emotional significance and therapeutic potential in such contexts.

## 5 Conclusions

This article proposes a care model sequence of three actionable frameworks: triggering, imagining, and responding. Caregivers can use this model to turn robotic interaction into a practical, intuitive tool. This is a new method of training caregivers to implement and adapt robotic technologies as part of routine care. This sequence has the potential to form the basis of robot-assisted therapy, enhancing human-to-human engagement by capturing attention, stimulating memories.

Current models largely depend on therapists or external experts to operate robots, but this is not scalable. Given the global shortage of care staff, empowering everyday caregivers to use off-the-shelf robots in structured, therapeutic sessions is essential. By leveraging available shelf-products robots, this approach offers a scalable and cost-effective solution for health and care organizations, particularly in the context of MCI and dementia. It provides caregivers with practical tools and a clear framework to optimize robot use. The study emphasizes that success lies not just in selecting the right robots, but in understanding how to use them effectively to enhance communication, engagement, and emotional wellbeing.

## Data Availability

The datasets presented in this article are not readily available because due to ethical requirements, we are unable to share data. Requests to access the datasets should be directed to Keren Mazuz, kerenma@jmc.ac.il.

## Footnotes

1 Retrieved from: https://www.btl.gov.il/English%20Homepage/Benefits/LongTerm%20Care/Services/Pages/day-care-centers.aspx (June 29, 2025). Day care pricing varies according to the benefit level the participant is granted according to Israel's Long-Term Care Insurance (LTCI) law (21).

2 Based on the Android version 8.1 operating system, the robot's is 19.5 cm in height, weighs approximately 360 grams. For more information about RoBoHoN see: https://robohon.com/.

3 As part of the larger research project, we developed an application intended to generate basic Hebrew conversation. However, significant technical challenges limited its use. For this study, the robot was therefore used in its standard off-the-shelf mode, speaking only English. The technical aspects of this application fall outside the scope of the present article.

4 An iterative thematic analysis is a method for identifying, analyzing and reporting patterns (themes) within data. For more information about the iterative thematic analysis see: (41, 43).

5 Psychoanalytic object-relations theory conceives psychological life as an ever-shifting dialogue with “internal objects,” internalized representations of early attachment figures (26). So dementia symptoms signal threatened or disrupted access to these objects and the anxieties this loss provokes. Psychoanalytically-informed care therefore tasks caregivers with providing a containing, reflective presence that receives patients' projections and, through steady human contact, reverie, and expressive media, such as music or art, recreates a secure emotional environment that eases distress across all stages of dementia.

6 In that sense, it is important to note that Sobral et al. (37), explained the importance of interventions in social communications among older adults with MCI. These interventions may relate to improving sociability, social interactions and communication because anxiety and depression are common in MCI patients (12) and may limit expressive and receptive communication (42).
